# Chloride intracellular channel proteins respond to heat stress in *Caenorhabditis elegans*

**DOI:** 10.1371/journal.pone.0184308

**Published:** 2017-09-08

**Authors:** Jun Liang, Yakov Shaulov, Cathy Savage-Dunn, Stephane Boissinot, Tasmia Hoque

**Affiliations:** 1 Department of Science, Borough of Manhattan Community College / CUNY, New York, New York, United States of America; 2 Department of Biology, Queens College, CUNY, Flushing, New York, United States of America; 3 Biology PhD Program and Biochemistry PhD Program, the Graduate Center, New York, New York, United States of America; 4 New York University Abu Dhabi, Saadiyat Island campus, Abu Dhabi, United Arab Emirates; Louisiana State University Health Sciences Center, UNITED STATES

## Abstract

Chloride intracellular channel proteins (CLICs) are multi-functional proteins that are expressed in various cell types and differ in their subcellular location. Two CLIC homologs, EXL-1 (excretory canal abnormal like-1) and EXC-4 (excretory canal abnormal– 4), are encoded in the *Caenorhabditis elegans* genome, providing an excellent model to study the functional diversification of CLIC proteins. EXC-4 functions in excretory canal formation during normal animal development. However, to date, the physiological function of EXL-1 remains largely unknown. In this study, we demonstrate that EXL-1 responds specifically to heat stress and translocates from the cytoplasm to the nucleus in intestinal cells and body wall muscle cells under heat shock. In contrast, we do not observe EXC-4 nuclear translocation under heat shock. Full protein sequence analysis shows that EXL-1 bears a non-classic nuclear localization signal (NLS) that EXC-4 is lacking. All mammalian CLIC members have a nuclear localization signal, with the exception of CLIC3. Our phylogenetic analysis of the CLIC gene families across various animal species demonstrates that the duplication of CLICs in protostomes and deuterostomes occurred independently and that the NLS was subsequently lost in amniotes and nematodes, suggesting convergent evolution. We also observe that EXL-1 nuclear translocation occurs in a timely ordered manner in the intestine, from posterior to anterior regions. Finally, we find that *exl-1* loss of function mutants are more susceptible to heat stress than wild-type animals, demonstrating functional relevance of the nuclear translocation. This research provides the first link between CLICs and environmental heat stress. We propose that *C*. *elegans* CLICs evolved to achieve different physiological functions through subcellular localization change and spatial separation in response to external or internal signals.

## Introduction

Adapting to sudden temperature change is one of the fundamental requirements for cells to maintain cellular homeostasis, as well as for animal development and survival. Thermal stress is among the most ubiquitous stressors that organisms have to resist. Therefore, signaling pathways and proteins that regulate thermal stress are highly conserved across species [1–3].

Chloride intracellular channel proteins (CLIC) are multifunctional proteins homologous to the glutathione S-transferase family [4]. Mammalian CLICs have six members, which have conserved protein sequences including transmembrane domains and nuclear localization signals (NLS) [5–7]. The proteins exist in either water soluble form or integral membrane form [8,9]. Research on CLICs demonstrates a variety of functions among them. CLIC3 has been shown to affect invasion and metastasis through promoting integrin recycling in various cancers such as ovarian and breast cancer [10–12]. CLIC3 also plays an important role in fetal growth and development [13]. The X-linked *Clic2* gene was found to be associated with intellectual and developmental disability, as well as in regulating Ca^2+^ signaling and cardiac muscle contraction, [14–16]. CLIC4 regulates cellular stress, autophagy, cell cycle arrest, apoptosis, fibroblast differentiation, macrophage innate response, carcinogenesis, and angiogenesis [17–21]. It serves as an adaptor component during signal transduction through interacting with p53, c-myc, cytoskeleton proteins, and transcription factors [7,17,22–24]. It has been reported that CLIC4 nuclear translocation is induced by nitric oxide, starvation, and cellular stress agents such as DNA damaging agents, metabolic inhibitors, cytotoxic molecules, and TNF-α, [17,19,25–27]. Several lines of evidence support that nuclear CLIC4 is associated with growth arrest in normal tissue and contributes to tumor growth *in vivo* [27,28]. Most research on CLICs has been conducted in mammalian cell lines and primary cell culture systems. Therefore, CLIC’s physiological functions in intact live animals are poorly understood.

Given that vertebrate CLIC members are expressed in different cell types and functions in various cellular process, we asked whether CLICs functions are conserved in *C*. *elegans*, and whether we could identify any novel CLIC functions. The CLIC family in the nematode *C*. *elegans* provides a more amenable model to study the functional diversity of CLICs since its genome contains only two CLIC homologs: EXL-1 and EXC-4. To date, most studies in *C*. *elegans* have focused on *exc-4*, since the loss of function mutants display a cyst defect in the unicellular excretory canal, where the excretory cell lumen is disrupted by swelling [29–31]. Expression patterns of EXC-4 and EXL-1 are largely distinct, with exceptions in a few tissues [30]. For example, only EXC-4 is specifically targeted to excretory canal cell and seam cell. Only EXL-1, however, is targeted to body wall muscle and the PVD neuron. However, both proteins are co-expressed in the intestine and some neurons. No apparent abnormal phenotypes have been found in *exl-1* loss of function mutants [30], except that in conjunction with *exc-4* mutants (but not alone) response to acute ethanol is deficient [32]. To date, functions of *exl-1* are not well understood.

In this article, we report that *C*. *elegans* CLIC homolog EXL-1 translocates from the cytoplasm into the nucleus under heat stress. Supporting functional importance of this phenomenon, *exl-1* loss of function mutants have decreased heat resistance compared with wild-type animals. In the intestine, EXL-1 nuclear translocation starts at the posterior region and then slowly reaches to the anterior region. In addition, we demonstrate that the CLIC homolog EXC-4 did not translocate into the nucleus under heat stress. Sequence analysis shows that EXL-1, but not EXC-4, bears a nonclassic nuclear localization signal which further supports our subcellular localization findings. Our study indicates that CLIC homologs differ in physiological functions, at least in heat stress management. This research also revealed a novel function of CLICs, regulating environmental heat stress.

## Results

### EXL-1 translocates into the nucleus in both intestinal and body wall muscle cells specifically under heat stress, but not under oxidative stress

**Fig 1:**
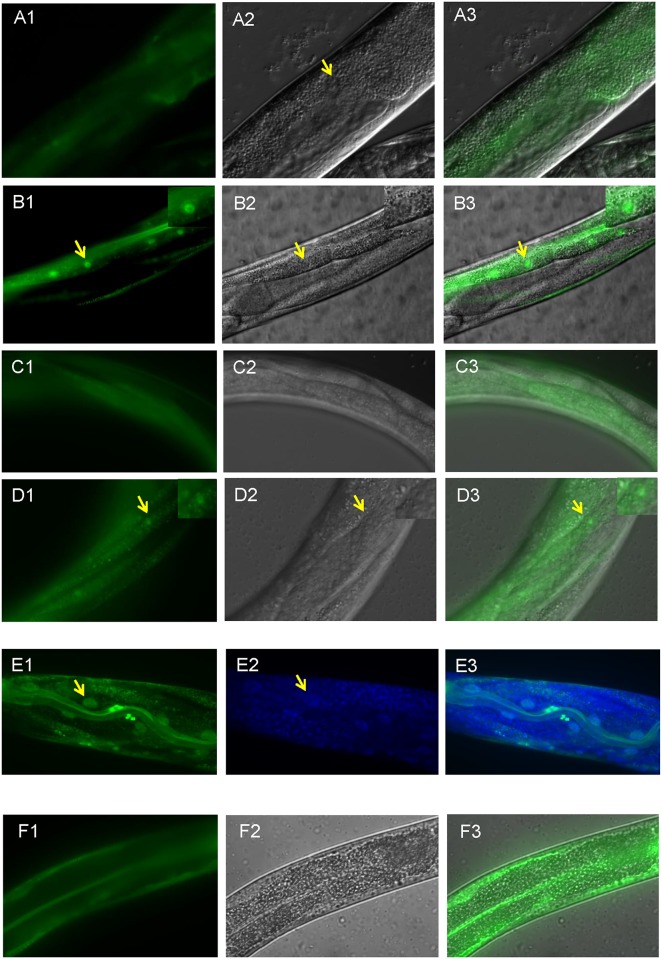
EXL-1 accumulates into the nucleus after heat shock. (A1-A3): EXL-1::GFP is expressed in intestinal cells at standard culture condition (20°C). The protein is diffuse inside the cells. No exclusion of nuclear expression was observed. (B1-B3): EXL-1::GFP accumulates into the intestinal nuclei (arrows) when animals were subjected to heat shock at 35°C for 2 hours. Inset shows enlarged image of the nucleus. (C1-C3): EXL-1::GFP is expressed in body wall muscle cells. (D1-D3): EXL-1::GFP translocates into the nucleus in body wall muscle cells (arrows) under heat shock at 35°C for 2 hours. Inset shows enlarged image of the nucleus. (E1-E3): DAPI staining in L4 animals shows that EXL-1::GFP signal overlaps with DNA staining inside the nucleus. (F1-F3): EXL-1::GFP does not translocate into the nucleus under 200mM paraquat treatment for 4 hours. Images are taken at L4 stage. 1: GFP fluorescence image; 2: Nomarski image (E2: DAPI staining); 3: merged image.

To examine whether EXL-1 subcellular localization changes under heat stress, integrated lines expressing an EXL-1::GFP fusion protein under native *exl-1* promoter in wild type N2 background were generated. Strong fluorescence was observed in various cell types including intestinal cells and body wall muscle cells at standard culture temperature (20°C) (Fig 1A and 1C), which is consistent with previous studies [30]. The expression is diffuse, mostly cytoplasmic inside the intestinal cells (Fig 1A). We next tested whether EXL-1 localization changed upon heat stress. Interestingly, we observed that cytoplasmic EXL-1::GFP translocates into the nucleus in both intestine cells and body wall muscle cells under heat shock (Fig 1B and 1D). Similar results were observed at 32°C and 37°C (data not shown). EXL-1::GFP expression in intestinal cells appears at L3 (third larvae) and later stages, but is barely visible at earlier developmental stages. Consistently, EXL-1::GFP nuclear translocation was also observed when animals reach to L3 stage and older, but not at any embryonic stage, L1 (first larvae), and L2 (second larvae) stages. To confirm EXL-1::GFP cellular location after heat shock, we used DAPI to stain DNA inside the nucleus (Fig 1E). There is a clear overlap between DAPI and GFP signals. Thus, EXL-1::GFP is translocated inside the nucleus upon heat shock. Furthermore, we quantitated nuclear to cytoplasmic fluorescence ratio, which is 1.79±0.06 after 2 hour heat shock at 35°C. Taken together,our data suggests that EXL-1/CLIC changes its subcellular localization in response to heat stress.

Next, we asked whether EXL-1 responds to other stress cues in *C*. *elegans*. We tested oxidative stress by using paraquat, a widely used oxidative reagent [33]. However, we did not observe any EXL-1::GFP nuclear translocation including both intestinal and body wall muscle cells, even with paraquat concentration as high as 200mM for 4 hours, where most animals were dead after the treatment (Fig 1F; data not shown under 100mM paraquat treatment). Thus, *C*. *elegans* EXL-1 responds to heat stress, but not oxidative stress, to change its subcellular localization.

**Fig 2:**
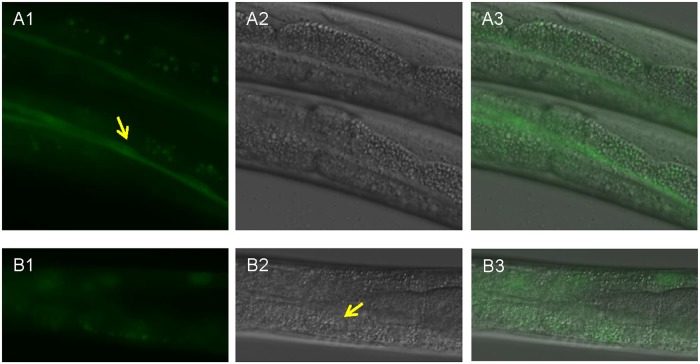
EXC-4 does not accumulate into the nucleus upon heat shock. (A1-A3): At 20°C, EXC-4::GFP is targeted to seam cells (arrow in A1) and intestinal cells. (B1-B3): Upon heat shock at 35°C for 2.5 hours, EXC-4::GFP did not accumulate into the intestinal nuclei (arrow in B2).

Then, we asked whether the other *C*. *elegans* CLIC homolog EXC-4 responds to heat stress in a similar manner. Integrated *exc-4*::*gfp* lines in wild type background were generated. Fluorescence was observed in various cell types including intestine and seam cells at 20°C (Fig 2A), which is consistent with previous studies [30]. Surprisingly, EXC-4::GFP did not translocate in the nucleus in the intestinal cells (Fig 2B). Therefore, our study demonstrates that EXL-1 and EXC-4 respond differently to heat stress, as observed by protein subcellular localization.

### EXL-1, but not EXC-4, bears a nonclassic nuclear localization signal (NLS)

**Fig 3:**
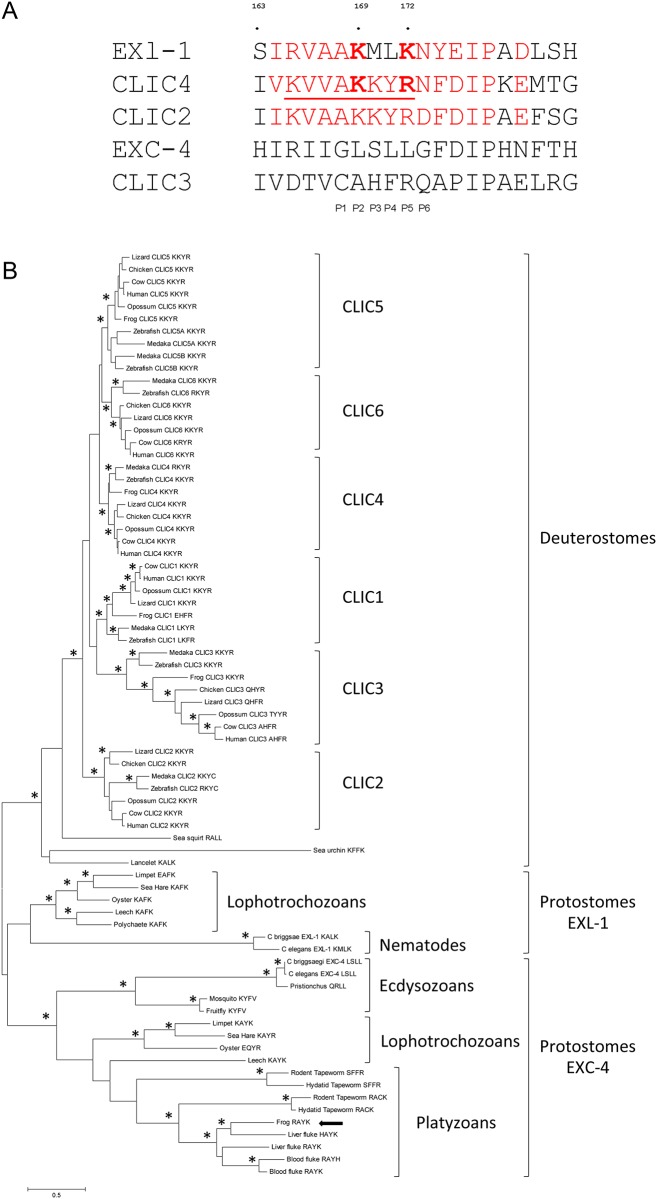
Sequence analysis of CLICs. (A): A putative NLS sequence is identified in EXL-1 protein sequence, but not in EXC-4. All human CLICs have a NLS motif except CLIC3 (shown here CLIC2 and CLIC4). EXL-1, CLIC2, and CLIC4 share conserved residues Lys at P2 (second binding position to importin-α) and Lys/Arg at P5. Both EXC-4 and CLIC3 sequences, however, lack NLS. Conserved amino acids are in red. NLS from CLIC are underlined. Two amino acids at P2 and P5 are bolded (B): Phylogenetic relationships among animal CLIC proteins. The tree was built using the maximum likelihood method. Nodes with higher than 70% bootstrap support are indicated with a star.

To better understand this subcellular localization change of EXL-1 and EXC-4 at the molecular level, we analyzed the protein sequences. A classic NLS is composed of clusters of basic residues arranged in either monopartite (one cluster of basic residues, such as NLS of SV40 large T antigen PKKKRKV) or bipartite (two clusters of basic residues separated by 10–12 residues) groups [34–36]. NLS-containing peptides bind to heterodimeric importin-α and -β in order to be transported through the nuclear pore into the nucleus [35]. A crystal structure of importin-α with the NLS (198 VKVVAKKYRN207) from CLIC4 shows that residues AKKYRN (an atypical NLS sequence, also referred to as binding position P1-6) contribute to peptide binding between CLIC4 and importin-α [6] and that motif KKYR is critical to bring the protein into the nucleus. Mutagenesis of KKYR into ITYG renders CLIC4 unable to translocate into the nucleus [25]. The key electrostatic interaction occurring between CLIC4 Lys203 (P2) and importin-α Asp192, Lys203 provides substantial energetic contribution to peptide binding and is critical to nuclear import [37]. In CLIC4, Arg 206 (P5) is typically occupied by a Lys residue in other NLS [6]. In EXL-1 sequence, 164IRVAAKMLKNY174 shares high sequence homology with corresponding CLIC4 sequence, where Lys169 is identical to CLIC4 Lys203 and Lys172 is a classic P5 residue in NLS (Fig 3A). In CLIC4, the bulky Tyr 205 makes favorable interactions with importin-α backbone. In EXL-1 sequence, the bulky Tyr 173 is three residues down from P4, which may also contribute significantly to interaction with importin-α. However, in EXC-4, we identified neither classic nor atypical NLS (Fig 3A). Taken together, we propose that EXL-1, but not EXC-4, bears a nonclassic NLS. This may explain our finding that EXL-1 translocates into the nucleus upon heat stress, whereas EXC-4 does not.

Interestingly, a nuclear localization signal (NLS) resides in all mammalian CLICs members except CLIC3 [6]. To examine whether absence of NLS in CLICs only occurs in *C*. *elegans* and mammals and understand the evolutionary history of CLIC gene families, we conducted a phylogenetic analysis using full length CLIC protein sequences from a variety of species representative of the major animal lineages, including vertebrates (medaka, zebrafish, frog, chicken, lizard, opossum, cow and human), non-vertebrate deuterostomes (sea squirt, sea urchin and lancelet), ecdysozoans (with the nematodes *C*.*elegans*, *C*.*briggsae*, and *Pristionchus pacificus* and two arthropods the fruitfly and the mosquito), lophotrochozoans (with three molluscs, oyster, sea hare, and limpet, and two annelids, a leeche and a polychaete) and Platyzoans (including two cestods, the rodent tapeworm and the hydatid tapeworm, and two trematods, the liver fluke and the blood fluke) (Methods).

The tree demonstrates that all CLIC members found in human are highly conserved in vertebrates (Fig 3B). Fish, reptile, amphibians, birds, and mammals all have six copies with the exception of possible loss of CLIC2 and CLIC6 in frog and CLIC1 in chicken. All vertebrate CLIC members form monophyletic and well-supported clades on the tree. However, the non-vertebrate deuterostomes species only have one copy in their genome. Thus, the duplications of the six CLIC copies occurred before the divergence of vertebrates, and are vertebrate-specific.

Nuclear localization motif KKYR found in human CLIC4 is highly conserved among CLIC1, 2, 4, 5, and 6, indicating that KKYR is most likely the ancestral state in vertebrates. There are however some minor modifications of the motif such as: CLIC1 has EHFR in frog and LKY/FR in fish, CLIC2 has K/RKYC in fish, CLIC4 has RKYR in medaka, CLIC6 has RKYR in zebrafish and KRYR in cow (Fig 3B). Since R and K residues possess similar biochemical features, modified motifs in CLIC4 and CLIC6 could still facilitate nuclear translocation.

The duplication of CLICs in protostomes (clade EXL-1 and clade EXC-4 on Fig 3B) occurred independently from the duplication in vertebrates. This duplication is very ancient and predates the split between ecdysozoans and lophotrochozoans. Following duplication, both anthropods and flatworms have lost the EXL-1 copy, thus the two duplicates are only observed in nematodes, molluscs, and annelids. Clade EXC-4 was further duplicated in cestods and trematods. Interestingly, a frog cDNA is grouping with the flukes. It is most likely that the frog cDNA sample was contaminated with its fluke parasite because a BLAT search of the frog genome with this cDNA sequence failed to recover any significant hit.

Fig 3B shows that CLIC3 in all vertebrate species, excluding fish species and frog, lacks nuclear localization motif KKYR motif. This suggests that loss of KKYR motif in CLIC3 is amniote specific. *C*.*elegans* EXC-4 also lacks the nuclear localization motif as shown in Fig 3A and supported by our experiments (Fig 2). The phylogenetic tree supports that loss of the NLS motif occurs independently in CLIC3 and in the *C*. *elegans* EXC-4.

### Inactivation of *exl-1* decreases thermotolerance under heat shock conditions

**Fig 4:**
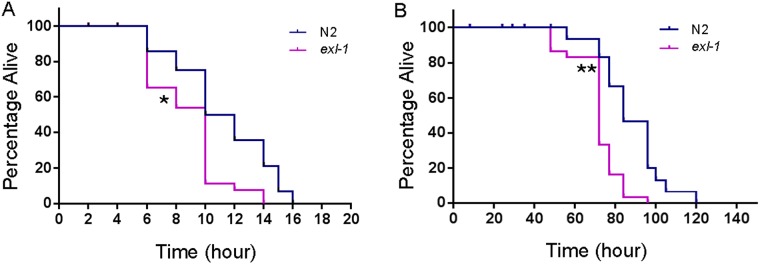
*exl-1* loss of function mutants are more susceptible to heat stress than wild type animals. (A): Animals were synchronized to young adult stage at 20°C and then were subjected to heat shock at 35°C. *exl-1* loss of function mutants are more sensitive to heat stress than wild type animals. (B): Animals were subjected to heat shock at 32°C. *exl-1* loss of function mutants lived significantly shorter than wild type animals. (Log-rank test was used to determine statistic power. * p value < 0.01; ** p value < 0.001)

We then asked, what is the physiological function of *exl-1*? Does it regulate animal response to heat stress? *exl-1(ok857)* is a null mutant due to a 650bp deletion, which removes a100bp upstream promotor region, exon 1, and part of exon 2 [30]. At 35°C, wild-type animals live to a maximum of about 16 hours, while *exl-1* loss of function mutants live to a maximum of about 14 hours (Fig 4A). Median survival of wild type is 11 hours, and that of *exl-1* mutants is 10 hours. Statistic analysis showed that the difference is significant (Log-rank test p value < 0.01). Similar results were observed at 32°C, where median survival of wild type is 84 hours, while that of *exl-1* mutants is 72 hours (Fig 4B). The survival difference is significant based on Log-rank test (p value < 0.001). In addition, overexpression of exl-1 leads to resuced thermotolerance at 32°C (S1 Fig), suggesting that the levels of EXL-1 must be tightly regulated. Thus, our data supports that gene *exl-1* regulates heat stress management in intact animals.

### EXL-1::GFP nuclear translocation in intestinal region occurs in an ordered manner

**Fig 5:**
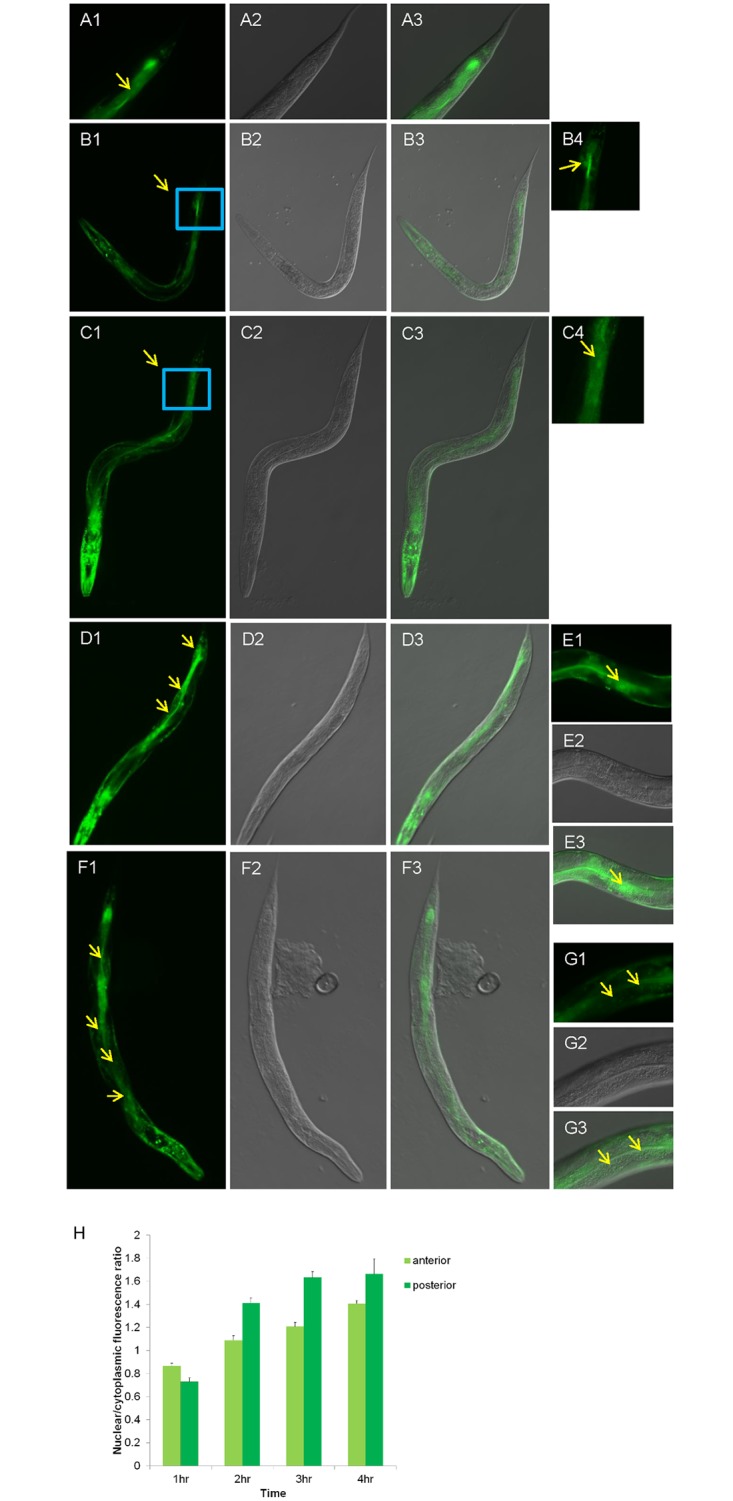
EXL-1::GFP nuclear translocation in intestine occurs in an ordered manner. (A1-A3): At 20°C, EXL-1::GFP has basal nuclear accumulation at the most posterior region (arrow in A1). (B1-B4): After heat shock at 35°C for 1 hour, EXL-1::GFP nuclear accumulation still remains in the most posterior region. B4 is enlarged image of the rectangular part of B1. (C1-C4): After 2 hour of heat shock, EXL-1::GFP nuclear translocation mostly occurs between middle region (around vulva) and tail region. C4 is enlarged image of the rectangular part of C1. (D1-D3): After 3 hours of heat shock, EXL-1::GFP nuclear translocation appears in the middle region and posterior region. (E1-E3): Enlarged images show EXL-1::GFP nuclear translocation in the middle region of intestine after 3 hours of heat treatment. (F1-F3): After 4 hours of heat shock, EXL-1::GFP nuclear translocation was observed in the whole intestinal region from anterior to posterior. (G1-G3): Enlarged images of EXL-1::GFP nuclear translocation at anterior region of intestine after 4 hours of heat treatment. Image A1-3, B4, C4, E1-3, G1-3 are taken with 400 magnification power; all the other images are taken with 100 magnification power; (H): Quantification of nuclear to cytoplasmic fluorescence ratio under different heat shock treatments.

At 20°C, EXL-1::GFP is evenly distributed inside the cells. We did not observe any exclusion of EXL-1::GFP from the nucleus, rather a diffuse expression across the whole cell (Fig 1A). A basal level of EXL-1::GFP nuclear accumulation at most posterior region of the intestine (near the tail) was observed at 20°C (Fig 5A). This might due to the pointed round structure of the animal tail, where EXL-1::GFP nuclear localization signal is enriched in a narrower region compared with the rest of the intestine.

Interestingly under heat stress, the EXL-1::GFP nuclear translocation in the intestine starts at the posterior region, and then slowly extends to the anterior region. At the beginning of heat treatment at 35°C for about 2 hours, most of EXL-1::GFP nuclear translocation was observed at the posterior region of the intestine between the middle (around vulva) and the tail end, almost none was observed at the anterior region (Fig 5C). Then, after 3 hours of heat treatment EXL-1::GFP nuclear translocation was clearly and predominantly observed in the middle and posterior regions of the intestine, but weakly at the anterior region (Fig 5D and 5E). After 4 hours of heat treatment, EXL-1::GFP nuclear translocation was observed across the whole intestine (Fig 5F and 5G). Thus, EXL-1 response to the heat signal occurs in the posterior region of intestine at first, then spreads to the middle, and ultimately reaches to the anterior region. However, EXL-1 nuclear translocation in body wall muscle cells was rather dramatic and rapid; a timely order has not been observed. Quantification of nuclear to cytoplasmic fluorescence signal also support this phenomenon (Fig 5H).

## Discussion and conclusion

Two CLIC homologs exist in the *C*. *elegans* genome, which makes the nematode *C*. *elegans* an excellent model to study functional diversification through evolution, as well as to identify new roles of the family members in animal physiology [30].

This research for the first time linked a CLIC family member with heat stress. We report here that EXL-1 and EXC-4 respond differently to thermal stress, in particular subcellular localization change. EXL-1 and EXC-4 co-localize in intestinal cells [30]. Only EXL-1, not EXC-4, is expressed in body wall muscle cells. In this study, we show that under heat shock conditions, EXL-1 is translocated into the nucleus in both intestinal cells and body wall muscle cells; while EXC-4 does not translocate into the nucleus in intestinal cells (Figs 1 and 2). Our data also demonstrate that EXL-1 nuclear translocation occurs under heat stress (Fig 1). Mammalian member CLIC4 also translocates into the nucleus under various conditions as stated above [19,25,26], thus CLICs share some similarity in cellular function across divergent organisms. Nuclear CLIC4 has been associated with pro-apoptotic activity, growth arrest, and tumor growth [17,25,27,28]. *In vivo*, CLIC4 is nuclear in multiple normal epithelial tissues. Nuclear CLIC4 is lost in multiple human tumors. In xenograft model when nuclear-targeted CLIC4 was introduced, tumor growth was retarded [27]. Nuclear-targeted CLIC4 also causes apoptosis in various cell types [25]. We then asked, what is the physiological function of nuclear EXL-1?

It has been shown that the intestine is one of the important tissues for stress response [38–40]. Many stress-regulating genes are expressed in intestine such as *daf-16* [41], *skn-1* [42], *sir-2*.*1* [43], *yap-1* [44], *pmk-1* [44]. Our survival assays show that *exl-1* loss of function mutants are more susceptible to heat stress than wild type animals (Fig 4). At both 32°C and 35°C, *exl-1* loss of function mutants demonstrated a significantly decreased heat resistance (Fig 4). Thus, gene *exl-1* regulates animal heat management. As demonstrated above, at both heat shock conditions EXL-1 translocates into nucleus. It is possible that nuclear EXL-1 contributes to its heat resistance. Together, we propose that EXL-1 and EXC-4 play different roles in heat stress management probably through changing protein subcellular localization, even though they are co-expressed in the same tissue: intestine.

Sequence alignment of CLICs across animals demonstrated that duplication of CLICs in protostomes and deuterostomes occurs independently (Fig 3B). EXL-1, but not EXC-4, protein sequence displays a nonclassic NLS sequence (Fig 3A). This explains why only EXL-1, not EXC-4, translocates into the nucleus upon heat stress. Interestingly, vertebrate CLIC3 also lost the NLS, while the other CLICs have kept the sequence. Thus this may indicate a convergent evolution of CLICs across animal phyla. Mammalian *Clic3* plays an important role in fetal growth and development [13]. In comparison, *exc-4* loss of function mutants produce less progeny than wild type animals (unpublished observation). However, whether lacking of NLS contributes to embryonic development still needs further investigation.

Our study, together with previous research [30], demonstrates that *C*. *elegans* CLIC homologs differ in physiological functions, at least in heat stress management (this study) and excretory canal formation. It has been previously shown that *exc-4*, but not *exl-1*, functions in the excretory system. Loss of function of *exc-4* leads to disruption of excretory cell lumen, the cystic phenotype. However, *exc-4* promoter-driven *exl-1* successfully rescued cystic phenotype in *exc-4* mutants, suggesting that *exl-1* can substitute for *exc-4* function in excretory canal development if expressed in the proper cells. However, only *exc-4*, but not *exl-1*, is expressed in the excretory canal cell. Therefore, nematode CLICs are spatially separated into various tissues to achieve functional variation in excretory canal development [30]. Consequently we propose that nematode CLICs functional diversities are achieved by protein subcellular localization change and spatial separation in response to external signals or during normal animal development.

We observed that EXL-1 nuclear translocation in intestine under heat stress occurs in a timely ordered manner: starts from posterior and spreads to anterior region (Fig 5). To explain this phenomenon, one possiblity is that there are signals/molecules at animal tail region that directly promote EXL-1 nuclear translocation under heat stress. Alternatively, EXL-1 nuclear translocation is regulated by secondary signal of heat stress since EXL-1 nuclear translocation does not occur until heat stress at 35°C last around 1.5 ~ 2 hours. Thirdly, EXL-1 protein sequence and NLS sequence may also contribute to the rate of nuclear translocation. It has been reported that various NLS sequences differ significantly the import rate of cargo protein into the nucleus [36]. Further research is required to clarify this.

Gradient and polar signals have been long discovered in animal development, growth, and patterning, such as decapentaplegic (Dpp) gradient in the Drosophila wing imaginal disc [45], Hedgehog (Hh) in limb development [46,47]. It has been reported that a death fluorescence (DF) burst occurs right before *C*. *elegans* death [48], which typically originates in the anterior-most cells of the intestine and then spreads rapidly along the intestine in an anterior to posterior wave. It will be interesting to understand the underlying mechanism of EXL-1 nuclear translocation gradient formation (posterior to anterior) under thermal stress.

## Materials and methods

### Strains and chemicals

*C*. *elegans* strains were obtained from the Caenorhabditis Genetics Center and cultured using standard methods and grown at 20°C unless otherwise noted [49]. The following strains were used: N2 (wild type), *exl-1(ok857) II*, OH 2211 (*otEx1184*[P_exl-1_::exl-1::gfp + rol-6(su1006)]) [30], OH 1279 (*otEx671*[P_exc-4_::exc-4::gfp + rol-6(su1006)]) [30], *exl-1 (ok857)* II; qc Is52. We integrated *otEx1184* and *otEx671* by gamma radiation [50]. *qcIs52* is an integrated derivative of *otEx1184; qcIs58* is an integrated derivative of *otEx671*; The integrated lines were outcrossed twice before the experiment. *exl-1 (ok857)* were outcrossed at least 4 times before conducting experiments.

### Microscopic imaging

Animals were immobilized by 10μl 25mM NaN_3_ in agarose pad for imaging under Zeiss AX10 fluorescence microscope [51]. Image was taken by Zeiss AxioCam Camera digitally. The whole animal image was taken by 10x objective lens, while portion of animal body image was taken by 40x objective lens. Integrated *exl-1*::*gfp* and *exc-4*::*gfp* line in N2 background were incubated at 35°C for 1–4 hours to observe protein subcellular localization. Animals before heat shock treatment serve as control group. Ratio of nuclear and cytoplasmic fluorescence is quantified by Image J (NIH).

### Heat shock

Animals were well-fed and cultured at 20°C for at least two generations before experiments. Animals were synchronized to fourth larval (L4) stage by egg laying method as described [51]. Briefly, 10–12 adult animals were placed in worm plates and laying eggs for 4–5 hours; then remove all the mothers; eggs were allow to hatch and grow at 20°C for approximately 48 hours to reach to L4 stage (proper L4 stage were confirmed by visualizing vulva structure under microscope), then subject those L4 animals to heat shock at 35°C. At least three independent trials were performed for each experiment.

### Oxidative stress

Animals were synchronized to L4 as stated above. We then washed off the worms by M9 buffer and clean the animals by M9 buffer three time to remove bacteria. Paraquat (Sigma-Aldrich) was added to the worm suspension in M9 to a final concentration of 100mM and 200mM respectively. Then we incubate them at 20°C for 4 hours and subject animals for imaging.

### DAPI staining

L4 animals after heatshock treatment were picked into M9 on a glass slide; then remove M9; animals were subject to 95% ethanol fixation for three times. After fixation, 10ug/ml DAPI (from Biotium) was used to stain DNA in the whole organism.

### Phylogenetic analysis

CLIC homologs were collected from Genbank and are either predicted sequences from complete genome or cDNA sequences. The list of sequences with their accession number is available as supporting information (S1 Table). The protein sequences were manipulated and aligned with CLUSTAL-W using the Geneious 8.1.5 platform (created by Biomatters and available at www.geneious.com). The phylogenetic tree was built using the maximum likelihood method implemented in MEGA 5.0 [52]. The optimal mutation model was determined to be the LG+G+I model by the Bayesian Information Criterion. The robustness of the nodes was assessed using a bootstrap procedure with 1,000 replicates.

### Survival assay

Animals were synchronized to L4 stage as described [51]. Then 30 L4 animals were picked for each strain to fresh worm plates. After 12–20 hours, those animals reach to young adulthood (stage was verified by visualizing 2–4 eggs retained inside uterus). We then subject those young adult animals under heat shock, at 32°C or 35°C respectively. At 32°C, number of live and dead animals was counted for three times per day until all animals were dead. At 35°C, number of live and dead animals was counted for every 2 hours until all animals were dead. Individuals that do not respond to a gentle prodding with a platinum wire will be scored as dead. Log-rank (Mantel-Cox) test is used for statistical analysis (GraphPad Prism 6).

## Supporting information

S1 Fig Animals were subjected to heat shock at 32°C. Integrated *exl-1*::*gfp* in wildtype N2 background animals displayed a severe heat sensitivity (p value < 0.0001, compared with N2). Inactivation of endogenous *exl-1* in the *exl-1*::*gfp* background significantly improve animal’s thermo tolerance (p value = 0.001, *exl-1(exl-1*::*gfp)* compared with *N2(exl-1*::*gfp*). We used egg-laying method to synchronize animals to day 1 young adults, then subject them to heat shock assay. For each experiment, 45 animals were used. Log-rank (Mantel-Cox) test is used for statistical analysis (GraphPad Prism 6). * p < 0.05; *** p < 0.0001 compared with N2.
(TIF)

S1 Table The list of CLIC homologs sequences used with their accession number from Genebank. (DOCX)
