# Supplementary figures and images for: Chloride intracellular channel proteins respond to heat stress in Caenorhabditis elegans
Source: PLoS One. 2017 Sep 8;12(9):e0184308. doi: 10.1371/journal.pone.0184308 (PMC5590911; doi:10.1371/journal.pone.0184308)

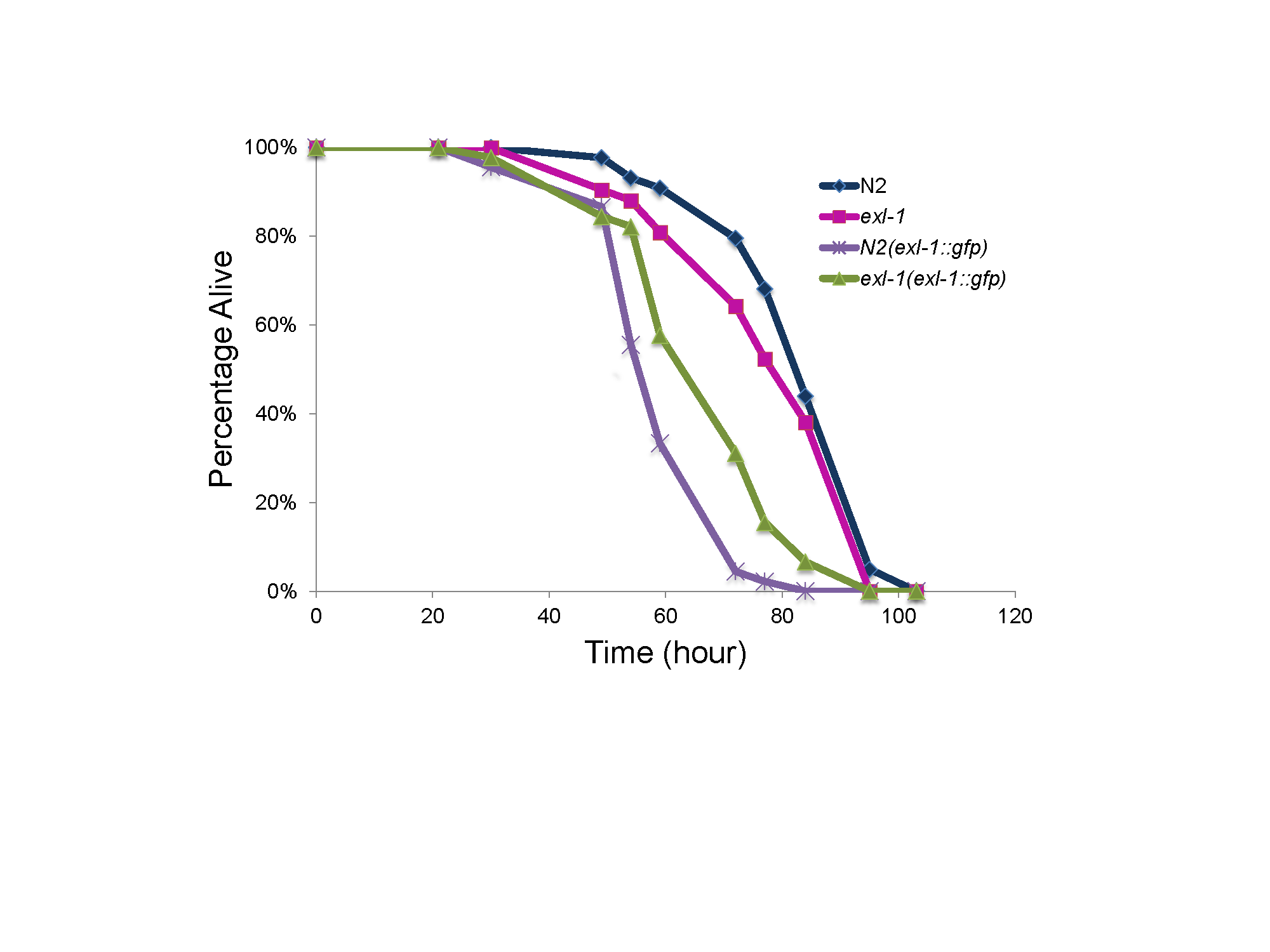

Supplement: S1 Fig — Integrated exl-1::gfp in wildtype N2 background animals displayed a severe heat sensitivity (p value < 0.0001, compared with N2). Inactivation of endogenous exl-1 in the exl-1::gfp background significantly improve animal’s thermo tolerance (p value = 0.001, exl-1(exl-1::gfp) compared with N2(exl-1::gfp). We used egg-laying method to synchronize animals to day 1 young adults, then subject them to heat shock assay. For each experiment, 45 animals were used. Log-rank (Mantel-Cox) test is used for statistical analysis (GraphPad Prism 6). * p < 0.05; *** p < 0.0001 compared with N2. (TIF) [file pone.0184308.s001.tif]
