# Supplementary material for: Chloride intracellular channel proteins respond to heat stress in Caenorhabditis elegans
Source: PLoS One. 2017 Sep 8;12(9):e0184308. doi: 10.1371/journal.pone.0184308 (PMC5590911; doi:10.1371/journal.pone.0184308)
Supplement: S1 Table — (DOCX) [file pone.0184308.s002.docx]

| CLIC protein | Accession number | Species latin name | Species Common name |
| --- | --- | --- | --- |
| CLIC1 | NP_001274522 | *Homo sapiens* | human |
| CLIC1 | NP_001015608 | *Bos taurus* | cow |
| CLIC1 | XP_007483623 | *Monodelphis domestica* | opossum |
| CLIC1 | XP_003228103 | *Anolis carolinensis* | lizard |
| CLIC1 | AAH59765 | *Xenopus tropicalis* | frog |
| CLIC1 | XP_004073822 | *Oryzias latipes* | medaka |
| CLIC1 | NP_997847 | *Danio rerio* | zebrafish |
| CLIC2 | NP_001280 | *Homo sapiens* | human |
| CLIC2 | NP_001075196 | *Bos taurus* | cow |
| CLIC2 | XP_007506978 | *Monodelphis domestica* | opossum |
| CLIC2 | XP_008119342 | *Anolis carolinensis* | lizard |
| CLIC2 | NP_001026285 | *Gallus gallus* | chicken |
| CLIC2 | XP_004073139 | *Oryzias latipes* | medaka |
| CLIC2 | NP_001002561 | *Danio rerio* | zebrafish |
| CLIC3 | NP_004660 | *Homo sapiens* | human |
| CLIC3 | NP_001093771 | *Bos taurus* | cow |
| CLIC3 | XP_007475465 | *Monodelphis domestica* | opossum |
| CLIC3 | XP_008121594 | *Anolis carolinensis* | lizard |
| CLIC3 | AAI21650 | *Xenopus tropicalis* | frog |
| CLIC3 | XP_003642297 | *Gallus gallus* | chicken |
| CLIC3 | XP_004075016 | *Oryzias latipes* | medaka |
| CLIC3 | NP_955818 | *Danio rerio* | zebrafish |
| CLIC4 | NP_039234 | *Homo sapiens* | human |
| CLIC4 | NP_001073687 | *Bos taurus* | cow |
| CLIC4 | XP_001366884 | *Monodelphis domestica* | opossum |
| CLIC4 | XP_003227259 | *Anolis carolinensis* | lizard |
| CLIC4 | AAH80344 | *Xenopus tropicalis* | frog |
| CLIC4 | XP_417741 | *Gallus gallus* | chicken |
| CLIC4 | XP_004082435 | *Oryzias latipes* | medaka |
| CLIC4 | AAH51622 | *Danio rerio* | zebrafish |
| CLIC5 | NP_058625 | *Homo sapiens* | human |
| CLIC5 | NP_776701 | *Bos taurus* | cow |
| CLIC5 | XP_007484066 | *Monodelphis domestica* | opossum |
| CLIC5 | XP_008114885 | *Anolis carolinensis* | lizard |
| CLIC5 | AAH76899 | *Xenopus tropicalis* | frog |
| CLIC5 | XP_004935978 | *Gallus gallus* | chicken |
| CLIC5A | XP_004066623 | *Oryzias latipes* | medaka |
| CLIC5A | NP_001007386 | *Danio rerio* | zebrafish |
| CLIC5B | XP_004083390 | *Oryzias latipes* | medaka |
| CLIC5B | AAI62229 | *Danio rerio* | zebrafish |
| CLIC6 | NP_444507 | *Homo sapiens* | human |
| CLIC6 | XP_583818 | *Bos taurus* | cow |
| CLIC6 | XP_001376468 | *Monodelphis domestica* | opossum |
| CLIC6 | XP_003219068 | *Anolis carolinensis* | lizard |
| CLIC6 | XP_425551 | *Gallus gallus* | chicken |
| CLIC6 | XP_004086085 | *Oryzias latipes* | medaka |
| CLIC6 | XP_009303556 | *Danio rerio* | zebrafish |
| CLIC-Deuterostome | XP_002129137 | *Ciona intestinalis* | sea squirt |
| CLIC-Deuterostome | XP_001178018 | *Strongylocentrotus purpuratus* | sea urchin |
| CLIC-Deuterostome | XP_002598862 | *Branchiostoma floridae* | lancelet |
| EXL1 | NP_497000 | *Caenorhabditis elegans* | nematode |
| EXL1 | CAP30321 | *Caenorhabditis briggsae* | nematode |
| EXL1 | ESO11341 | *Helobdella robusta* | leech |
| EXL1 | ELU12613 | *Capitella teleta* | polychaete worm |
| EXL1 | XP_011444438 | *Crassostrea gigas* | oyster |
| EXL1 | ESO82544 | *Lottia gigantea* | limpet |
| EXL1 | XP_005089773 | *Aplysia californica* | sea hare |
| EXC4 | XP_002640719 | *Caenorhabditis briggsae* | nematode |
| EXC4 | NP_740950 | *Caenorhabditis elegans* | nematode |
| EXC4 | KKA73574 | *Pristionchus pacificus* | nematode |
| EXC4 | XP_003437150 | *Anopheles gambiae* | mosquito |
| EXC4 | AGB95379 | *Drosophila melanogaster* | fruitfly |
| EXC4 | ESO94842 | *Lottia gigantea* | limpet |
| EXC4 | XP_012942275 | *Aplysia californica* | sea hare |
| EXC4 | EKC30516 | *Crassostrea gigas* | oyster |
| EXC4 | ESO03698 | *Helobdella robusta* | leech |
| EXC4 | CDS32292 | *Hymenolepis microstoma* | rodent tapeworm |
| EXC4 | CDS21611 | *Echinococcus granulosus* | hydatid tapeworm |
| EXC4 | CDS33797 | *Hymenolepis microstoma* | rodent tapeworm |
| EXC4 | EUB60614 | *Echinococcus granulosus* | hydatid tapeworm |
| EXC4 | AAH93565 | *Xenopus laevis* | frog / fluke |
| EXC4 | XP_009176414 | *Opisthorchis viverrini* | liver fluke |
| EXC4 | XP_009173057 | *Opisthorchis viverrini* | liver fluke |
| EXC4 | XP_012795085 | *Schistosoma haematobium* | blood fluke |
| EXC4 | CAX71023 | *Schistosoma japonicum* | blood fluke |
